# Growth mindset and well-being in social interactions: countering individual loneliness

**DOI:** 10.3389/fpubh.2024.1368491

**Published:** 2024-06-24

**Authors:** Chao Wang, Shanshan Li, Yilin Wang, Mengxia Li, Weidong Tao

**Affiliations:** ^1^School of Teacher Education, Huzhou University, Huzhou, China; ^2^Department of Psychology, Neuroscience and Behaviour, Faculty of Science, McMaster University, Hamilton, ON, Canada; ^3^School of Medicine and Nursing, Huzhou University, Huzhou, China

**Keywords:** growth mindset, loneliness, interpersonal distress, well-being, social interactions

## Abstract

**Introduction:**

Loneliness is a prevalent negative emotion experienced by college students. This study explores the relationship between a growth mindset and loneliness among college students.

**Methods:**

A total of 560 college students completed the Growth Mindset Scale (GMS), UCLA Loneliness Scale (UCLA), Interpersonal Relationships Assessment Scale (IRS), and two measures assessing distinct facets of well-being the Satisfaction with Life Scale (SWLS) and the revised Positive Affect and Negative Affect Scale (PANAS).

**Results and discussion:**

The results found a significant negative correlation between a growth mindset and loneliness. A growth mindset negatively predicted loneliness through the chain-mediated effects of interpersonal distress and well-being. These findings underscore the important role of a growth mindset in influencing loneliness, providing teachers and practitioners a new perspective to understand and intervene college students’ psychological challenges.

## Introduction

Loneliness is an unpleasant subjective experience that occurs when an individual’s desired social relationships do not align with the quantity or quality of their actual social network (1). It is a key factor posing a threat to individual’s mental health (2). Previous research suggests that the age of 20 is the first peak period for experiencing loneliness (3). As college students navigate their independent lives, factors such as their cognitive processes, interpersonal relationships, and well-being contribute significantly to their experience of loneliness (4–6). Prolonged exposure to such negative emotions can impact an individual’s self-perception and increase the risk of depression (7). Therefore, investigating the factors contributing to loneliness is essential for identifying potential threats to the mental health of college students and establishing a foundation for implementing suitable preventive and intervention measures across all societal levels.

Mindsets, originally termed “implicit ideas,” refer to a core assumptions about the malleability of individual traits (8), forming a framework for interpreting and responding to adversity (9, 10). Dweck (11) categorized mindsets into growth mindsets and fixed mindsets. Existing research has demonstrated that a growth mindset enhances the development of an individual’s non-cognitive competencies, positively influencing emotional competencies such as psychological resilience and mental well-being (10, 12), and mitigating negative emotional perceptions including depression and anxiety (13). Interpersonal problems are significant for mental health protection and are key determinants of well-being and loneliness (14). Further investigation into the effects of growth mindset on interpersonal distress could clarify its role in social interactions.

The purpose of this study is to examine the influence of a growth mindset on individual loneliness from a social interaction perspective and to clarify the potential mechanisms through the major factors of interpersonal distress and well-being. This study will have implications for improving educational strategies at home and in schools, aiming to increase college students’ resilience against loneliness.

### Growth mindset and loneliness

A growth mindset profoundly influences the development of an individual’s social and emotional competences (12, 15), suggesting that intelligence and abilities can be improved through learning (9). Previous studies have examined the impact of a growth mindset on learning literacy and engagement from a cognitive perspective (16, 17). More recently, increased attention has shifted toward the influence of a growth mindset on emotional and affective experiences (12, 15). Individuals who adopt a growth mindset believe in the plasticity of the brain, embrace challenges, respond optimistically to failures, and exhibit enhanced psychological stress regulation (9, 18). A growth mindset strongly predicts positive emotions and is significantly correlated with well-being (12). In contrast, a fixed mindset is often associated with negative emotions such as shame, anxiety, and depression (19). These findings suggest that a growth mindset can negatively predict the presence of negative emotions.

Loneliness is a prevalent mental health concern among college students (20, 21) and a primary cause of anxiety and depression during interpersonal interactions (22). Despite its significant impact on an individual’s mental health and academic development (23), few studies have directly explored the influence of a growth mindset on loneliness. There is a need to further explore how a growth mindset shapes the experience of loneliness among college students. Therefore, this study proposes Hypothesis 1: A growth mindset is significantly correlated with loneliness.

### The mediating role of interpersonal distress

Interpersonal distress is characterized by negative emotional experiences such as anxiety, loneliness, and depression that arise from challenges in real social interactions (24). Individuals experiencing interpersonal distress often struggle to regulate their relationships positively, tending toward withdrawn and an avoidant attitude toward life (25). This suggests that interpersonal distress is a significant barrier to successful social interaction and positive psychological development.

Previous studies show that individuals’ emotional experiences and behaviors are significantly influenced by their mindsets (15, 26, 27). Those with a growth mindset, in particular, are noted for their enhanced openness-related traits such as curiosity and creativity (28), and superior emotion regulation in the face of challenges (29). Additionally, interpersonal distress can exacerbate feelings of loneliness as it hinders the formation of positive social connections and the fulfillment of intimate needs (30).

A review of the literature indicates that although many studies have examined interpersonal distress in conjunction with social–emotional competencies such as psychological resilience, attachment, and negative emotions, relatively few have explored it from the perspective of mindsets. Moreover, the role of interpersonal distress as a mediating variable in the relationship between mindsets and loneliness has been largely overlooked. In light of these findings, the study proposes Hypothesis 2: Interpersonal distress mediates the relationship between a growth mindset and loneliness among college students.

### The mediating role of well-being

Well-being is the overall evaluation and emotional experience of an individual’s life as they compare their actual life with their ideal life (31). It comprises a cognitive component — the overall evaluation of life quality and degree of satisfaction, i.e., life satisfaction — and an emotional component, which is the sum of various positive and negative emotions experienced, i.e., emotional well-being (32).

Howell (33) regarded a growth mindset as foundational in the study of well-being, considering it a defining characteristic of an individual’s ability to experience positive emotions. A growth mindset is a positive belief that exerts a protective influence on personal development, guiding individuals to adopt adaptive strategies when confronting mental problems, adversity and obstacles (34, 35). According to self-determination theory, relationships are also an important source of happiness. Relationship needs are one of the three intrinsic needs of an individual, and their fulfillment has a significant impact on well-being (36).

There is a constant interaction between well-being and loneliness (6). Well-being plays a pivotal role in mitigating loneliness. A greater sense of well-being is associated with improved perceptions of support and life satisfaction, which in turn lead to a reduction in loneliness (6). Therefore, Hypothesis 3 posits that well-being mediates the relation between a growth mindset and loneliness.

### The chain mediating role of interpersonal distress and well-being

**Figure 1:**
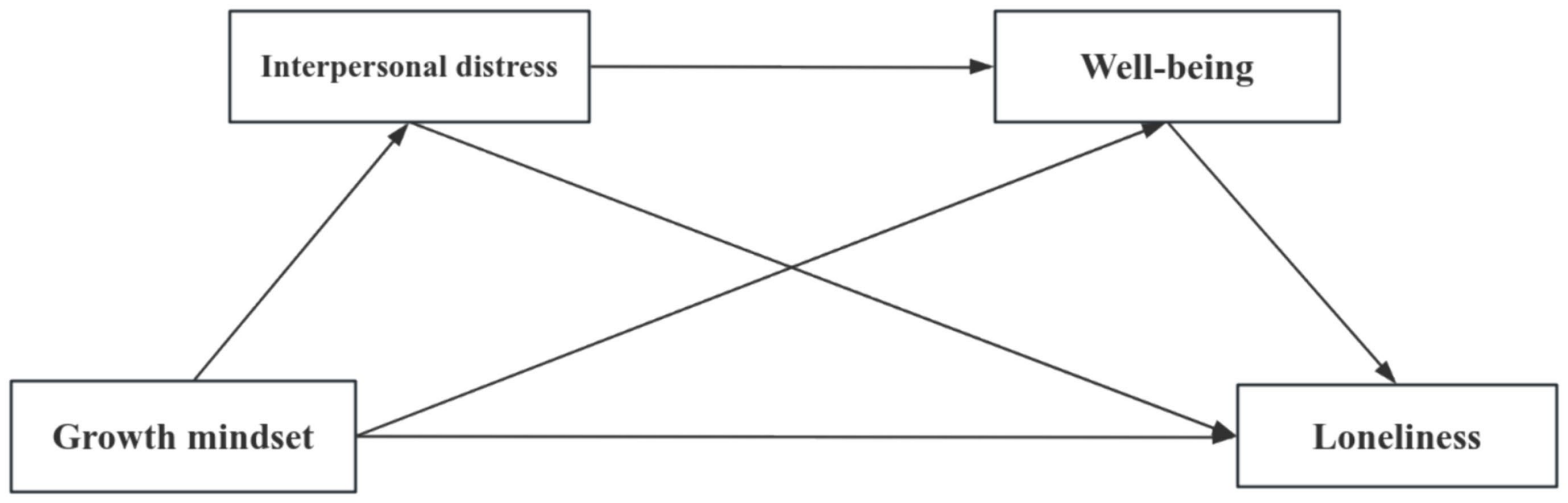
Hypothetical model of the chain mediation in this study.

The research discussed above demonstrates that interpersonal distress and well-being may mediate the pathways through which a growth mindset affects loneliness. Social interaction, as a crucial aspect of personal development, plays an essential role in forming close relationships and preserving emotional well-being (37, 38). It has been found that interpersonal distress negatively predicted well-being. Interpersonal distress can heighten an individual’s negative emotions, resulting in psychological stress, including anxiety and depression, which in turn can reduce well-being (39). This decrease in well-being due to interpersonal distress can ultimately increase an individual’s perceived loneliness. According to the above analysis, this study proposes Hypothesis 4: Interpersonal distress and well-being serve as sequential mediators between a growth mindset and loneliness among college students. Therefore, based on existing research, this study constructs a chain mediation model (see Figure 1) to explore the mechanism of how growth mindset affects loneliness.

## Methods

### Participants

The participants in this study were recruited through random cluster sampling of undergraduates at a Chinese university in May 2023. Participants were informed about the purpose of the questionnaire, confidentiality, and other related matters, and consent was obtained from all respondents. A total of 560 questionnaires were distributed, and after excluding those deemed invalid based on predefined criteria—such as incomplete submissions, responses where all checkboxes were the same, or responses in a recognizable pattern—527 valid questionnaires were collected. The effective response rate was 94.1%. Of the participants, 166 were male (31.5%) and 361 were female (68.5%). The average age of the participants was 18.36 years, with an age range from 18 to 21 years.

### Measures

#### Growth mindset

The Chinese version of the Growth Mindset Scale (GMS) was adapted from Dweck’s (11) original scale. The scale comprises six questions, evenly divided between forward-scoring (e.g., “You can always change your intelligence to a great extent.”) and backward-scoring items (e.g., “Intelligence is hard to change.”). Respondents used a 6-point scale, ranging from 1 (‘strongly disagree’) to 6 (‘strongly agree’). During data analysis, scores for the three reverse-scored questions were reversed, and the average score of all six questions was calculated. A higher score indicates a stronger growth mindset. In this study, the Cronbach’s *α* for the scale was found to be 0.883.

#### Loneliness

Loneliness was assessed based on the third edition of the UCLA Loneliness Scale, developed by Russell et al. (40). The scale comprises 20 questions, with 9 items being reverse-scored (e.g., “Do you often feel like you belong among friends?”). A 4-point scale was utilized, with higher scores indicating greater levels of loneliness. The internal consistency of the scale in this study was high, with Cronbach’s *α* of 0.894.

#### Interpersonal distress

The Interpersonal Relationships Assessment Scale (IRS), developed by Zheng (41), is widely used in China to assess interpersonal problems. The scale consists of 28 questions, with 12 positive-scoring and 12 negative-scoring questions, organized into four dimensions: making friends (e.g., “Feeling uncomfortable about meeting strangers”), communication (e.g., “Difficulty with continuous talks”), interacting with the opposite sex (e.g., “Too little interaction with the opposite sex”), and treating others (e.g., “Excessive envy and jealousy toward others”). A two-point scale was utilized, where a “yes” response was assigned 1 point and a “no” response was assigned 0 points. Higher scores indicate more severe interpersonal distress. In this study, the Cronbach’s alpha coefficient of the scale was 0.873, and the Cronbach’s alpha coefficients of each dimension were 0.701, 0.745, 0.633, and 0.669, respectively.

#### Well-being

Two instruments were used to measure the distinct facets of well-being. The Satisfaction with Life Scale (SWLS), developed by Diener et al. (42), consists of 5 questions (e.g., “I’m satisfied with my life”) with a 7-point scale ranging from “strongly disagree” to “strongly agree”. The revised Positive Affect and Negative Affect Scale (PANAS) includes 12 items with a 7-point scale to assess participants’ emotions (43, 44). In this study, the assessment of well-being followed procedures employed in prior studies (45, 46). Initially, the three scores were standardized, and then the aggregated score was calculated as the sum of Satisfaction with Life plus Positive Affect minus Negative Affect. Higher scores indicate greater perceived well-being. The Cronbach’s alpha coefficient for the scale ranged from 0.899 to 0.901.

#### Research procedures and data processing

The questionnaire was administered anonymously to the entire teaching class, following a standardized set of instructions. It took approximately 15 min for participants to complete the questionnaires, and responses were uniformly collected on-site. The collected data from the questionnaires were collated and analyzed using SPSS 26.0.

## Results

### Common method bias test

This study conducted Harman’s one-factor test, revealing that there were 16 factors with eigenvalues exceeding 1. The variability explained by the first factor was 21.24%, falling short of the 40 percent threshold. These results suggest that the issue of common method variance in this research was not significant.

### Descriptive statistics and correlation analysis for each variable

This study examined the correlation between variables using Pearson correlation analysis and identified significant relationships. The results found a significant negative correlation between a growth mindset and both loneliness and interpersonal distress. Additionally, a significant positive correlation was found between a growth mindset and well-being. Loneliness was significantly positively associated with interpersonal distress. Well-being was significantly negatively associated with loneliness and interpersonal distress. Detailed results are presented in Table 1.

**Table 1:** Description statistics and correlation analysis.

	*M ± SD*	1	2	3	4
1. Growth mindset	3.29 ± 0.93	-			
2. Loneliness	2.15 ± 0.45	−0.235^**^	-		
3. Interpersonal distress	0.32 ± 0.20	−0.228^**^	0.618^**^	-	
4. Well-being	0.20 ± 10.66	0.190^**^	−0.601^**^	−0.451^**^	-

^**^p < 0.01.

### Mediator model

Firstly, the study sequentially tested the significance of the regression coefficients of growth mindset → loneliness, growth mindset → interpersonal distress → well-being → loneliness in turn. Then, the study analyzed the chain mediation effect of variables using the PROCESS macro program in SPSS. Given the significant correlation between personal ability, economic status, and other variables, the analysis was conducted with the former two factors as control variables. In this analysis, growth mindset was considered the independent variable, while interpersonal distress and well-being were treated as mediator variables, loneliness as the dependent variable.

The results of the regression analyses, as shown in Table 2, revealed that a growth mindset had a significant negative predictive impact on interpersonal distress (*β* = −0.189, *p* < 0.001). Interpersonal distress had a significant negative predictive impact on well-being (*β* = −0.382, *p* < 0.001). Well-being was a strong negative predictor of loneliness (*β* = −0.392, *p* < 0.001), while interpersonal distress was a significant positive predictor of loneliness (*β* = 0.423, *p* < 0.001). A growth mindset was found to be a significant negative predictor of loneliness (*β* = −0.064, *p* < 0.05), thereby supporting hypothesis 1.

**Table 2:** Regression analysis of mediating roles of interpersonal distress and well-being in the relationship between growth mindset and loneliness.

Outcome (Y)	Predictors (X)	*R* ^2^	*F*	*β*	*SE*	*t*	LLCI	ULCL
Regression 1								
Interpersonal distress	Growth mindset	0.106	20.776	−0.189	0.009	−4.501^***^	−0.058	−0.023
Regression 2								
Well-Being	Growth mindset	0.251	43.732	0.075	0.446	1.896	−0.031	1.723
	interpersonal distress			−0.382	2.149	−9.521^***^	−24.682	−16.238
Regression 3								
Loneliness	Growth mindset	0.517	111.432	−0.064	0.015	−2.007^*^	−0.061	−0.001
	Interpersonal distress			0.423	0.080	12.119^***^	0.810	1.124
	Well-being			−0.392	0.001	−11.147^***^	−0.020	−0.014
Regression 4								
Loneliness	Growth mindset	0.109	21.220	−0.201	0.020	−4.790^***^	−0.137	−0.058

*N* = 527. Bootstrap sample size = 5,000. CI, confidence interval. ^*^p < 0.05, ^***^p < 0.001.

**Figure 2:**
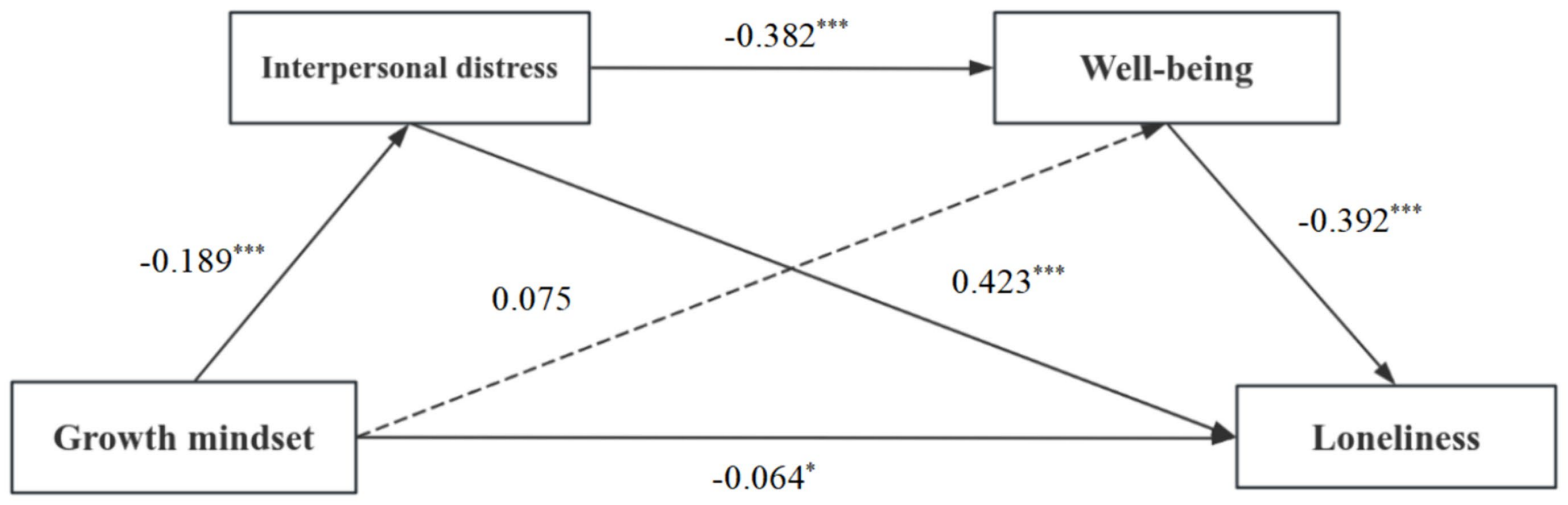
Conceptual model of the chain mediation.

Mediation effect analyses revealed that interpersonal distress and well-being significantly mediated the relation between growth mindset and loneliness, with a standardized mediation effect value of −0.067. The mediation effect consisted of two pathways: indirect effect 1, formed from growth mindset to interpersonal distress to loneliness (effect value: −0.039), confirming hypothesis 2 and indirect effect 2, from growth mindset to interpersonal distress to well-being to loneliness (effect value: −0.014), validating hypothesis 4. Detailed results are shown in Table 3 and visually represented in Figure 2.

**Table 3:** Total, direct and indirect effects in Chain mediation analysis.

	Effect	Boot *SE*	BootLLCI	BootULCI	
Total Ind	−0.097	−0.020	−0.137	−0.058	
Direct Ind	−0.031	0.015	−0.061	−0.001	
Indirect Ind	−0.067	0.016	−0.098	−0.037	69.07%
G → D → L	−0.039	0.010	−0.059	−0.020	40.21%
G → W → L	−0.014	0.009	−0.032	0.002	
G → D → W → L	−0.014	0.004	−0.023	−0.007	14.43%

G, growth mindset; L, loneliness; D, interpersonal distress; W, well-being.

## Discussion

To investigate the internal mechanisms underlying the effects of a growth mindset on loneliness among college students, our study constructed a chain mediation model to analyze the sequential mediating roles of interpersonal distress and well-being. Our findings indicate that a growth mindset is negatively associated with interpersonal distress, which, in turn, is negatively associated with well-being. The negative prediction of loneliness by college students’ growth mindset is mediated through interpersonal distress. Additionally, our results show that growth mindset predicts loneliness through the sequential mediation of interpersonal distress and well-being. By exploring the influence of a growth mindset on loneliness from an interpersonal perspective, this study not only enriches our understanding of how loneliness emerges among college students but also provides a deeper insight into the complex interplay of individual psychological traits and their social outcomes. The implications of these findings offer valuable directions for developing targeted interventions aimed at alleviating loneliness and enhancing the mental health of college students.

### Growth mindset significantly predicts loneliness

The present study found that a growth mindset significant negatively predicts loneliness. This finding is consistent with previous studies, which has shown that growth mindset training can mitigate the impact of loneliness on an individual’s academic performance (23). Mindsets shape individuals’ emotional, behavioral, and physiological responses to stress and adversity (9). Accordingly, individuals with a growth mindset are better equipped to cope with emotional stress and demonstrate greater proficiency in managing mental health issues, such as anxiety and depression (13, 47). Furthermore, possessing a growth mindset is positively associated with the use of cognitive reappraisal, a key emotion regulation skill (15). These findings underscore the crucial role of a growth mindset in not only understanding but also intervening in the development of loneliness. The negative association revealed between a growth mindset and loneliness highlights the potential for growth mindset interventions to serve as effective strategies for reducing loneliness among college students.

### The mediating role of interpersonal distress

Mindsets influence individuals’ goal selection and behavioral tendencies in interpersonal relationships (26, 27), and these relationships are key predictors of one’s perceived loneliness (5). The present study demonstrated that interpersonal distress mediates the relationship between a growth mindset and loneliness. This may be attributed to the fact that individuals with a growth mindset strive for a sense of self-worth and believe in the continuous enhancement of their abilities (48). As a result, individuals tend to proactively address interpersonal issues, leading to reduced interpersonal distress and the cultivation of close social relationships. This finding is consistent with previous research, which has shown that a growth mindset is significantly positively correlated with the quality of interpersonal relationships (49). Additionally, Markovic et al. (50) found that the link between shyness and internalized negative coping was twice as strong in adolescents with a fixed mindset compared to those with a growth mindset in situations of peer-related frustration.

Simultaneously, individuals with a growth mindset exhibit better psychological resilience (51) and can promptly adapt to the psychological stress caused by interpersonal distress, counteracting the feelings of loneliness arising from such distress. Simcharoen et al. (52) investigated the relationship between loneliness and interpersonal distress and found that interpersonal distress positively predicted loneliness. Importantly, this study contributes to understanding the potential mechanism through which a growth mindset influences loneliness, specially by introducing interpersonal distress as a mediating variable.

### Chain mediation of interpersonal distress and well-being

While previous research has focused on the influence of individual variables on relationships or well-being in relation to loneliness (6, 53), this study demonstrates that interpersonal distress and well-being jointly serve as mediators between a growth mindset and loneliness. Social connection is theorized to be a fundamental human need, playing a crucial role in well-being (54). Studies have shown that positive relationships, such as those with parents, teachers, and peers, predict subjective well-being (55, 56). This implies that heightened levels of loneliness among college students may be attributed to elevated levels of interpersonal distress. This distress, in turn, could result from the absence of a growth mindset in effectively addressing the various challenges arising from social interactions and the associated psychological stresses, ultimately diminishing well-being (57, 58). This finding is in line with previous studies indicating that individuals with a growth mindset are better equipped to regulate their mindset in stressful situations, which is negatively associated with psychological distress (59, 60). Lelkes (61) also found that friendships positively predict well-being, mainly due to their positive and supportive effects.

In contrast, interpersonal distress worsens interpersonal stress and social anxiety (62, 63), leading to psychological distress and impacting well-being (64). If individuals are unable to fulfill their emotional needs in social relationships, it adversely affects their sense of well-being, making them more susceptible to loneliness. Therefore, harmonious nature of interpersonal relationships and the experience of well-being play an important role in the influence of mindsets on loneliness.

This study unveils the underlying mechanism through which a growth mindset influences loneliness, shedding light on the direct and indirect contributions of individual factors to the experience of loneliness among college students. It provides a fresh perspective on practical interventions for emotional issues among college students, suggesting that adopting a growth mindset can alleviate feelings of loneliness. A growth mindset can help college students in coping with interpersonal distress by enhancing their resilience in problem-solving and perceive well-being. Thus, this study offers valuable insights for enhancing college students’ well-being and sustaining emotional health through the cultivation of a growth mindset. Future educational practices could consider incorporating positive psychology approaches to assist college students in preventing and coping with loneliness by promoting a growth mindset.

### Limitations

However, several limitations should be acknowledged. Firstly, this study focused on the situational condition of interpersonal relationships and did not fully consider other factors that may contribute to loneliness. Future research should explore a broader array of scenarios. Second, the cross-sectional design of this study does not allow us to observe how the variables develop over time. Addressing this limitation would involve incorporating longitudinal studies in future research.

## Conclusion

This study extends the investigation into the mechanisms through which growth mindsets influence loneliness by exploring the sequential mediating roles of interpersonal distress and well-being. Addressing loneliness requires a heightened focus on developing social interaction abilities. Fostering a growth mindset in college students might be a valuable approach. Educators should be mindful of their language tendencies, reduce evaluations of individual abilities and stereotypes, and emphasize the value of hard work, guiding individuals in actively and positively coping with interpersonal distress. This proactive approach aids in fulfilling the need for intimacy in interactions and resisting loneliness.

## Data availability statement

The raw data supporting the conclusions of this article will be made available by the authors, without undue reservation.

## Ethics statement

The studies involving humans were approved by Huzhou University, School of Teacher Education. The studies were conducted in accordance with the local legislation and institutional requirements. The participants provided their written informed consent to participate in this study.

## Author contributions

CW: Conceptualization, Funding acquisition, Supervision, Writing – original draft, Writing – review & editing. SL: Conceptualization, Data curation, Formal analysis, Methodology, Visualization, Writing – original draft. YW: Conceptualization, Methodology, Validation, Writing – review & editing. ML: Data curation, Writing – review & editing, Supervision, Validation. WT: Conceptualization, Methodology, Supervision, Writing – review & editing.
